# *Emerging Themes in Epidemiology*: Form and function

**DOI:** 10.1186/1742-7622-1-2

**Published:** 2004-10-06

**Authors:** Ben A Lopman

**Affiliations:** 1 Deputy Editor, Emerging Themes in Epidemiology; 2 on behalf of the editorial board of Emerging Themes in Epidemiology; 3 Department of Infectious Disease Epidemiology, Imperial College London, London, United Kingdom

## Abstract

*Emerging Themes in Epidemiology *is a new, online Open Access journal. This editorial – which coincides with the Journal's launch – describes its unique review and publication model. The editorial board and review process of *ETE *will be managed by research degree students and will therefore be a training ground for students (though final editorial control rests with senior faculty Associate Editors). With our mix of Open Access publishing and the strong involvement of students in editing and reviewing, we believe that *Emerging Themes in Epidemiology *will be a progressive medium for promoting new ideas in epidemiology.

## 

The idea that has given birth to *Emerging Themes in Epidemiology (ETE) *was conceived late on a Friday afternoon. As the rest of the academic and professional work week was coming to an end, the creators of this journal were just beginning to think straight. Clearly the work of students. However, it was not just clandestine meetings at unsociable hours that brought this diverse group of epidemiology PhD students together to launch and direct a new journal.

Through training and various research projects PhD students become versed in the technical, methodological and practical aspects of epidemiology. While some will stay in academia and some will use their skills in other sectors of public health, our time as PhD students is seen as a time to learn and a time to be a part of the academic community. However, aside from the occasional correspondence with editorial boards, research students receive little training or involvement in the process of scientific publication. We think that this is an important process for us to learn about since, as researchers, our careers are dependent on publication. So we came together to form *ETE*, which will be a training ground for PhD students.

This Open Access journal will also be our contribution to the field of epidemiology. Students will manage the editorial board and they will be a part of the review committee for each paper. Through this process we hope to learn. But, we also hope to facilitate the publication of new ideas and to raise issues that concern us as students of epidemiology. In the other editorial in this launch 'issue' of *Emerging Themes in Epidemiology*, the philosophical underpinnings of the Journal have been illustrated. Now, some explanation is required of how we will achieve our vision of *ETE*.

## The Editorial Board

The editorial board of *ETE *is made up of doctoral students and faculty. The research students are from various academic and public health institutes in London (though we are open to the prospect of overseas expansion). The student members are responsible for the running of the Journal, for managing the review process and communicating with authors and readers. The board is headed by our Editor-in-Chief, Professor Peter Smith, and is overseen by an international panel of Associate Editors. The role of these senior faculty is to consider the major decisions of the Board and to preside over final publication judgements. And, because PhD studentship is a transient state (of varying length), these Associate Editors will maintain the continuity of *ETE *by recruiting new student editors as the current board graduates.

## The review process and student reviewers

At present, there is a lively debate as to whether peer-review should be 'open' or 'closed'. Recognizing the forceful arguments on both sides, we believe that the scientific community is best served by a system that encourages a constructive dialogue between colleagues. Authors and reviewers will know each others' name and affiliation. However, we recognize that some highly specialized fields of research are small and competitive. In reality, this means that there are often repercussions for giving both criticism and praise to a colleague's work. So at the reviewers' request, we will withhold their names from the authors.

When a paper is submitted to *ETE*, it is assigned to one of our two Deputy Editors. A committee of five research students then screens the manuscript to decide whether it fits within the scope of the Journal. If they decide it does, the paper is sent for external peer review. All papers are seen by a minimum of two reviewers. As part of our dedication to involving research students in the editorial process and facilitating the publication of new ideas, at least one PhD student and one expert in the appropriate field will be asked to review each paper. How will PhD students perform as reviewers? There is evidence that young reviewers (under 40 years old) trained in epidemiology or statistics, and those who can dedicate about 3 hours provide better quality reviews [1]. Clearly, these are characteristics of most of our student reviewers, so we are optimistic about our review process. Furthermore, in accord with our objective to make the Journal a training resource, all student reviewers will be informed of the Editorial Board's decision. They will see how their review influenced that decision and how it complemented or contradicted with the reviews of more experienced reviewers (the 'experts').

Final decisions to accept or reject will always be done with the endorsement of one of the Associate Editors, who are senior researchers in various epidemiologic fields. The involvement of students in the review process is admittedly novel, but we believe there are sufficient checks and balances in our review system to ensure quality. Testament to this is the decision by PubMed and PubMed Central to index *ETE*. Authors can therefore be confident that their papers will have the highest visibility and will be available in PubMed's archives.

## Conflict of interest in a journal dedicated to discourse

The issue of conflict of interest is anathema to many scientists. We are trained to pursue objectivity; the suggestion that one's methods, results or conclusions are in any way affected by funding sources or other affiliations can be seen as a direct challenge to credibility. It shouldn't be. In the complex funding and political environments in which we work, we can no longer deny that these issues must be faced. Evidence has mounted that funding is associated with research outcomes (in tobacco and food) and, recently, high-profile cases related to vaccine safety and nutritional supplements have furthered the case for explicit statements of potential conflicts [2-5]. Once again, *ETE *will encourage openness from authors as well as reviewers. An individual's financial or professional associations do not necessarily constitute conflicts of interest, but failing to declare them can be highly misleading to the reader and the scientific community [6].

*ETE *is a journal dedicated to active discourse in epidemiology. We will ask contributors to disclose their source(s) of funding – as it could be seen to influence opinions or research agenda. We will also ask authors to disclose links to other associations that are not explicit from their institutional affiliation.

## Open Access and financing of ETE

Perhaps there is no discipline more appropriate for open-access publication than epidemiologic research and the allied population health sciences. Our work has the aim of revealing the determinants of disease in human populations, with the ultimate goal, through knowledge empowerment, intervention and policy, to reduce suffering from ill health. With such an ethos, how can we justify keeping research findings locked away in expensive medical journals, inaccessible to those who may need it most?

The open-access publishing model has the potential to revolutionize the way authors use scientific literature and the manner in which it is received by other scientists and the community at large. The advent of open-access publishing means "it is now possible to make our treasury of scientific information available to a much wider audience, including millions of students, teachers, physicians, scientists, and other potential readers, who do not have access to a research library that can afford to pay for journal subscriptions" [7].

Currently, the most common economic model for sustaining Open Access journals is one where the author pays for the costs incurred in the editorial and publishing process. BioMed Central, our publisher, charges authors per accepted publication (currently $525), but fees are waived for authors from institutions with membership to BioMed Central or authors with financial hardship. Our hope is that a long-term solution will in future be developed by the scientific community as a whole, perhaps through the provision of grant schemes for Open Access publication. Nonetheless, recognizing the difficulties with the current 'author pays' model, we aim to cover the article processing fees for at least the next few years for all submissions not already covered under institutional memberships or other grants or subsidies.

## Special issues and online publishing

From time to time *ETE *will call for papers on a particular subject pertinent to the theory and practice of epidemiology. The series of articles will be built around a framework, designed by the editorial board and a guest editor. The series of articles will be published in tandem, and will be highlighted on a special page on the website. The flexibility of online publishing will allow for articles submitted after the initial publication of the issue and for commentary on the original articles to also be included. Guest editors for special issues will work with the Editorial Board to develop an issue, commission appropriate articles and write a leading editorial. Anyone interested in collaborating with *ETE *to curate a special issue is encouraged to contact the Editorial Board.

## The birth of ETE

Epidemiology is 'young' science, rapidly evolving in its theory, methods, and subject matter. With our mix of Open Access publishing and the strong involvement of students in editing and reviewing, we believe that *Emerging Themes in Epidemiology *will be a progressive medium for promoting new ideas that will contribute to the development of the field.

## References

[B1] Black N, van Rooyen S, Godlee F, Smith R, Evans S (1998). What makes a good reviewer and a good review for a general medical journal?. Jama.

[B2] Horton R (2004). The lessons of MMR.. Lancet.

[B3] White C (2004). Three journals raise doubts on validity of Canadian studies.. Bmj.

[B4] Levine J, Gussow JD, Hastings D, Eccher A (2003). Authors' financial relationships with the food and beverage industry and their published positions on the fat substitute olestra.. Am J Public Health.

[B5] Dyer C (1998). Tobacco company set up network of sympathetic scientists.. Bmj.

[B6] Smith R (2002). Making progress with competing interests. Bmj.

[B7] Brown PO, Eisen MB, Varmus HE (2003). Why PLoS Became a Publisher.. PLoS Biol.

